# Biodegradable MoSe_2_-polyvinylpyrrolidone nanoparticles with multi-enzyme activity for ameliorating acute pancreatitis

**DOI:** 10.1186/s12951-022-01288-x

**Published:** 2022-03-05

**Authors:** Pei Xie, Liying Zhang, Hui Shen, Hang Wu, Jiulong Zhao, Shige Wang, Lianghao Hu

**Affiliations:** 1 Department of Gastroenterology, Changhai Hospital, Naval Military Medical University, No. 168 Changhai Road, Shanghai, 200433 China; 2 Department of Chemistry, School of Materials and Chemistry, University of Shanghai for Science and Technology, No. 516 Jungong Road, Shanghai, 200093 China

**Keywords:** MoSe_2_-polyvinylpyrrolidone, Biodegradable nanoparticles, Reactive oxygen species, Acute pancreatitis, Nanozyme

## Abstract

Exogenous antioxidant materials mimicking endogenous antioxidant systems are commonly used for the treatment of oxidative stress-induced injuries. Thus, artificial enzymes have emerged as promising candidates for balancing and treating the dysregulation of redox homeostasis in vivo. Herein, a one-pot hydrothermal strategy for the facile preparation of MoSe_2_-polyvinylpyrrolidone (PVP) nanoparticles (NPs) is reported. The synthesized NPs were biodegradable due to their exposure to oxygen and exhibited high stability. Moreover, they effectively mimicked various naturally occurring enzymes (including catalase, superoxide dismutase, peroxidase, and glutathione peroxidase) and scavenged free radicals, such as 3-ethylbenzothiazoline-6-sulfonic acid, ·OH, ·O^2−^, and 1,1-diphenyl-2-picrylhydrazyl radical. Further apoptosis detection studies revealed that MoSe_2_-PVP NPs significantly increased the cell survival probability in H_2_O_2_ in a concentration-dependent manner. The cytoprotective effect of MoSe_2_-PVP NPs was explored for an animal model of acute pancreatitis, which confirmed its remarkable therapeutic efficacy. Owing to the biodegradable and biocompatible nature of MoSe_2_-PVP NPs, the findings of this work can stimulate the development of other artificial nanoenzymes for antioxidant therapies.

**Graphical Abstract:**

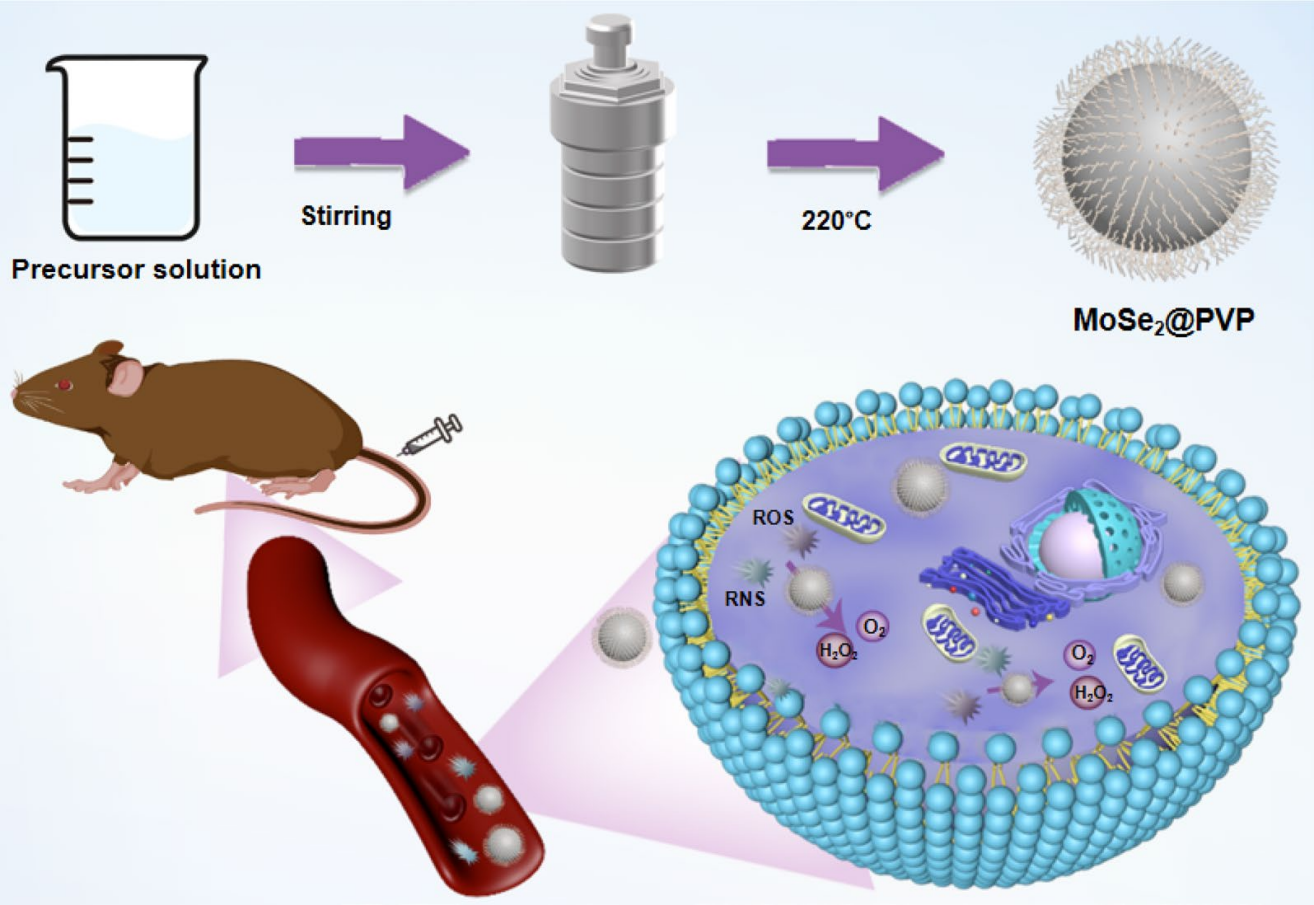

**Supplementary Information:**

The online version contains supplementary material available at 10.1186/s12951-022-01288-x.

## Introduction

Free radicals exist in different forms, including reactive oxygen species (ROS) (such as superoxide anion (·O^2−^), hydroxyl radical (·OH), and hydrogen peroxide (H_2_O_2_)) as well as reactive nitrogen species (RNS) such as nitric oxide (NO) [1–3]. Routine physiological activities of the human body produce large amounts of free radicals, and the free radical production and scavenging maintained their balance through a variety of mechanisms in the body [4]. Among them, natural enzymes have played an important role in the scavenging of free radicals [5]. Inflammation can activate epithelial cells, neutrophils, and macrophages to produce various inflammatory cytokines and other inflammatory mediators, which further reduce the expression of enzymes or impair their radical scavenging function [6–8]. Failure to scavenge free radicals leads to an oxidative stress response and breaks the free radical production-scavenging balance, which causes oxidative damage to proteins, DNA, and lipids and further accelerates the progression of inflammation [9–11]. Therefore, the prompt and effective scavenging of excessive free radicals plays a critical role in inflammation suppression [12–14].

Acute pancreatitis (AP) is a common digestive system disease with high incidence and mortality rate [15, 16]. AP is often accompanied by local tissue inflammations and the release of inflammatory mediators from overreacting leukocytes, causing a cascade response that leads to a systemic inflammatory response [17–19]. An increasing number of studies have suggested that oxidative stress plays a key role in AP progression [20, 21]. In particular, the excessive production of ROS and RNS causes oxidative damage to glandular follicle cells and regulates the transcription and transduction of redox-related pathways [4, 22, 23]. Therefore, antioxidants that can inhibit free radical production and scavenge free radicals may act as a potential clinical treatment for the RNS-mediated AP by reducing ROS levels [24]. Current AP treatments include various drugs such as bilirubin^25^, octreotide [26], and plant extracts [27, 28]. However, the preparation of these drugs requires conducting complex reaction processes, and their clinical application remains challenging [29, 30].

Artificial nanoenzymes are nanomaterials with enzymatic properties. Owing to their stability, small storage costs, and ease of synthesis on a large scale, artificial nanoenzymes have many advantages over natural enzymes [31–33]. Notably, certain types of nanoenzymes can mimic antioxidant systems in vivo, including superoxide dismutase (SOD), catalase (CAT), peroxidase (POD), and glutathione peroxidase (GPx) and efficiently scavenge harmful ROS and RNS to avoid the imbalance of oxidants and antioxidants [12, 34–36]. With the recent developments in nanomedicine, artificial nanozymes have been widely used for the treatment of inflammation-related diseases. Different types of nanomaterials, such as transition-metal dichalcogenides (TMDCs) [31, 37], framework-based nanoenzymes [38, 39], and precious metal nanomaterials [40, 41] and nanomaterials based composites [42, 43] were extensively studied as artificial nanozymes. For example, Prussian blue (PB) nanoparticles with multi-enzyme mimetic capabilities were effectively utilized for the ROS-mediated inflammatory bowel disease intervention [44]. In another study, a two-dimensional vanadium carbide (V_2_C) MXene nanoenzyme (MXenzyme) that mimicked a wide range of natural enzymes was used for inflammatory and neurodegenerative treatments [42]. These studies suggest the potential application of artificial enzymes in anti-inflammatory treatments. TMDCs exhibit a unique combination of atomic-scale thickness, direct bandgap, strong spin–orbit coupling and favorable electronic and mechanical properties, which make them interesting for fundamental studies in personalized medicine [45]. However, the possibility of using transition-metal dichalcogenides as artificial enzymes for AP treatment has not been studied in detail.

Herein, encouraged by the diverse applications of TMDCs in high-end electronics, spintronics, optoelectronics, energy harvesting, flexible electronics, DNA sequencing and personalized medicine [45], we prepared polyvinylpyrrolidone-modified molybdenum selenide nanoparticles (MoSe_2_-PVP NPs) for AP therapy. This single-component nanoenzyme is easy to synthesize, can mimic the intrinsic CAT, SOD, POD, and GPx systems, and possesses the ability to eliminate various ROS such as hydrogen peroxide, ⋅OH, and ⋅O^2−^. The fabricated MoSe_2_-PVP NPs exhibited high biocompatibility and biodegradability, and produced strong cytoprotective effects against the free radical-induced damage in vitro. The results of both Micro-CT scan and ICP indicated an enrichment of MoSe_2_-PVP NPs at the pancreatic site. The in vivo experiments revealed that MoSe_2_-PVP NPs demonstrated excellent therapeutic effects in a caerulein-induced AP inflammation model, which significantly downregulated the expression of inflammatory factors without causing adverse side effects.

## Experimental section

### Materials

All chemicals were used without further purification. (Trimethylol aminomethane hydrochloride) Tris–HCl, FeSO_4_·7H_2_O, salicylic acid (SA), and 2,2′-azino-bis (3-ethylbenzothiazoline-6-sulfonic acid) (ABTS) were purchased from Aladdin Bio-Chem Technology Co., Ltd. Sodium molybdate dihydrate (Na_2_MoO_4_, AR) and hydrazine hydrate (30%), Na_2_EDTA and Hydrogen peroxide (H_2_O_2_, 99%) were purchased from Sinopharm Chemical Reagent Co., Ltd. Polyvinylpyrrolidone (PVP, MW = 1300 k) was purchased from Beijing Bailingwei Technology Co., Ltd. Potassium persulfate (K_2_S_2_O_8_) was purchased from Shanghai Reagent Factory 2, Shanghai Chemical Reagent Factory. L-selenocysteine, 3,3',5,5'-tetramethylbenzidine (TMB, 98%), 1, 1-diphenyl-2-picrylhydrazyl (DPPH), 5,5-dimethyl-1-pyrroline N-oxide (DMPO), xanthine and xanthine oxidase were purchased from Shanghai Yuanye Bio-Technology Co., Ltd. Pyrogallol was purchased from Shanghai Titan Scientific Co., Ltd. Total glutathione peroxidase assay kit and DCFH-DA (2,7-dichlorofluorescein diacetate) reagent assay kit were purchased from Beyotime biotechnology Co., Ltd. Dulbecco's Modified Eagle Medium (DMEM) and phosphate buffer saline (PBS) were procured from Corning Co., Ltd. (Shanghai, China). The Cell counting kit-8 (CCK-8) was purchased from Dojindo Laboratories (Japan). Kunming (KM) mice (female, 4–6 weeks, 20–25 g) and C57BL/6 mice were ordered from Shanghai Slac Laboratory Animal Center (Shanghai, China). All experimental mice are bred strictly in accordance with the rules and regulations of the Minister of Health of the People's Republic of China (MOHC). Micro-CT(Life Medical Inc, Venus001)The Enzyme Linked Immuno Sorbent Assay (ELISA) Kits (TNF-α, IL-6, IL-1β) were obtained by Elabscience Biotechnology Co., Ltd. Calcein-AM/PI Live/Dead kit was purchased from Yi Sheng Biotechnology (Shanghai) Co., Ltd. Propidium iodide/Annexin V-FITC was purchased from Novizan Biotechnology Co., Ltd. Caerulein was purchased from MedChemExpress Co., Ltd.

### Synthesis of biocompatible MoSe_2_-PVP NPs

To synthesis the MoSe_2_-PVP nanoparticles by one-pot hydrothermal method, typically, under magnetic stirring, 0.1 g sodium molybdate dihydrate and 0.02 g L-selenocysteine were added to 25 mL of distilled water containing 0.1 g PVP (MW = 1300 k). After stirring for 20 min to form a homogeneous solution, 200 μL of hydrazine hydrate was then added. Finally, the above mixed solution was transferred into 100 mL polystyrene-lined stainless steel autoclave. After being reacted at 220 ^◦^C for 12 h and naturally cooled to room temperature, the precipitate was collected by centrifuge at 21,000 rpm for 15 min and washed 3 times with ethanol and deionized water. The product of MoSe_2_-PVP NPs was diluted to different concentrations and stored at 4 ^◦^C for further use.

### Characterizations of MoSe_2_-PVP NPs

The microstructure and size of MoSe_2_-PVP dispersed with alcohol were observed using transmission electron microscope (TEM, JEM-2100F) and scanning electron microscope (SEM, Zeiss Sigma 300). X-ray photoelectron spectroscopy (XPS, Thermo Scientific K-Alpha) and Fourier transform infrared spectroscopy (FTIR, Nicolet Nexus 470) was used to analyze the chemical valence and chemical bonds of MoSe_2_-PVP, respectively. The hydration diameters (DLS) of MoSe_2_-PVP NPs in varied dispersion medium were analyzed at various time points and the Zeta potential of MoSe_2_-PVP NPs were identified by Zetasizer Nanoseries system of Malvern Nano ZS90. Biodegradable properties was monitored by a UV–Vis spectrometer (Shimadzu, Japan).

### The biodegradability of MoSe_2_-PVP NPs

To investigate the degradation properties, we simulated the microenvironment of pancreatitis. The details are as follows: 1.5 mL, 0.2 mg/mL MoSe_2_-PVP NPs were dispersed in phosphate buffer solution (pH = 7.4) and packed into 2 mL centrifuge tubes and incubated in an incubator at 37 °C. Then, the absorbance value was measured by UV–Vis spectrometer (Shimadzu, Japan) and the photos before and after degradation were recorded.

### The overall antioxidant capacity of MoSe_2_-PVP NPs

The radical scavenging activities of the MoSe_2_-PVP NPs was studied using a ABTS strategy. Briefly, ABTS stock solution (7.4 mM) was mixed with K_2_S_2_O_8_ solution (2.6 mM) in the dark for 12 h to generate the ABTS radicals. Then, MoSe_2_-PVP NPs with different concentrations (0, 0.05, 0.10, 0.15, and 0.20 mg/mL) was added thereinto and cultured at 37 °C for 6 min. Moreover, MoSe_2_-PVP NPs with concentration of 0.20 mg/mL was cultured at 50 °C for 25 min and then added thereinto and cultured at 37 °C for 6 min. The absorbance of mixed solution at 752 nm was monitored via a UV–Vis spectrometer (Shimadzu, Japan). And the scavenging ratio were calculated as follows (Eq. 1):

1 $${\text{Scavenging ratio }}\left( {\text{\% }} \right){\text{ = 1{-}}}\frac{{\text{A}}}{{{\text{A}}_{{0}} }}{\text{ * 100\% }}$$

where A is the absorbance of control, and A_0_ is the absorbance of the sample.

### Scavenging activity of MoSe_2_-PVP NPs on H_2_O_2_

The characteristic absorption change of H_2_O_2_ at 240 nm was detected by UV–Vis spectrometer (Shimadzu, Japan) to evaluate the catalytic decomposition of H_2_O_2_ by MoSe_2_-PVP. Briefly, 20 mM H_2_O_2_ was mixed with 0.2 mg/mL MoSe_2_-PVP, and then the absorbance values at 240 nm were immediately detected as a function of time. Similarly, various concentrations of MoSe_2_-PVP (0, 0.05, 0.1, and 0.2 mg/mL) were incubated separately with 20 mM H_2_O_2_ at room temperature for 1 h. And then the concentration of remained H_2_O_2_ was then measured and the scavenging capacity of H_2_O_2_ was calculated as Eq. 2.

2 $${\text{Scavenging ratio }}\left( {\text{\% }} \right){ = }\frac{{{\text{A}}_{{1}} {\text{ - A}}}}{{{\text{A}}_{{1}} }}{\text{ * 100\% }}$$

Herein, A and A_1_ are the absorbance values of H_2_O_2_ solution with and without MoSe_2_-PVP NPs, respectively.

### Measurement of POD-like of MoSe_2_-PVP NPs

The POD-like ability of MoSe_2_-PVP was studied at room temperature using the TMB method. Typically, the reaction systems containing MoSe_2_-PVP (300 μL, 0.2 mg/mL), H_2_O_2_ (1000 μL, 10 mM), and TMB (0.6 M) were used to demonstrate the chromogenic reaction to imply the POD-like activity. The absorbance of the reaction system (652 nm for TMB) was recorded by UV–Vis spectrometer (Shimadzu, Japan). Meanwhile, the concentration of MoSe_2_-PVP NPs was changed (0, 0.1, and 0.2 mg/mL) to indicate the concentration-dependent POD-like ability.

### Scavenging activity of MoSe_2_-PVP NPs on·OH

The ·OH scavenging properties of MoSe_2_-PVP NPs were examined using the paramagnetic resonance spectroscopy (ESR, Bruker, Germany). Typically, 20 μL of 1 mM FeSO_4_·7H_2_O, 20 μL of 250 mM DMPO and 20 μL of 10 mM H_2_O_2_ were added to ultrapure water and mixed in a quartz capillary with or without the addition of different concentrations of MoSe_2_-PVP NPs solution (0, 0.4, and 0.8 mg/mL). The ESR spectrum was detected by capturing the signal of the spin adducts DMPO/·OH immediately. The ·OH scavenging properties of MoSe_2_-PVP NPs were further evaluated via the salicylic acid (SA) method. In the reaction system, FeSO_4_·7H_2_O (100 μL, 9 mM), ethanol-salicylic acid (100 μL, 9 mM), deionised water (1 mL), and MoSe_2_-PVP NPs (0, 0.05, 0.1, and 0.2 mg/mL) were added sequentially, and H_2_O_2_ (100 μL, 8.8 mM) was added and shaken well in a water bath at 37 °C for 30 min. The above solution was centrifuged and the supernatant was collected for absorbance measurement at 510 nm using a UV–Vis spectrometer (Shimadzu, Japan).

### Scavenging activity of MoSe_2_-PVP NPs on superoxide anions radicals

To verify the SOD-like ability of MoSe_2_-PVP NPs, ·O^2−^ was generated through the xanthine (5 mM) and 10 μL xanthine oxidase (0.1 U mL^−1^) in 50 mM PBS, and added DMPO to form an adduct DMPO/·OOH-, and then MoSe_2_-PVP NPs at various concentrations (0, 0.4, and 0.8 mg/mL) were added into the reaction system for ESR test. The pyrogallol method was also used to study the SOD-like ability of MoSe_2_-PVP NPs. Tris–HCl (50 mM) containing Na_2_EDTA (2 mM), MoSe_2_-PVP NPs (0, 50, 100 or 200 μg/mL) and 1,2,3-trihydroxybenzen (5 mM) were immediately mixed and poured into the cuvette and measured at 320 nm for 5 min at 25 °C.

### Measurement of GPx-like activity of MoSe_2_-PVP NPs

For assessment of the GPx-like activities, the total glutathione peroxidase assay kit with NADPH (Beyotime, Shanghai, China) was used. According to the instruction, the detection buffer of glutathione peroxidase, MoSe_2_-PVP NPs solution (0.05, 0.1, and 0.2 mg/mL), NADPH (62.5 mM), glutathione (GSH, 75 mM), GR and the GPx working solution were mixed and added to a 96-well plate and incubated for 15 min at 25 °C to exclude oxidized glutathione (GSSG) from interfering with the subsequent assay. Then, peroxide reagent (10 µL, 30 mM) solution was added to the well plate and mix well. The microplate reader (SpectraMax) was used for continuous absorbance determination (340 nm, 5 min). Finally, the GPx enzyme activity was calculated (Eq. 3).

3 $$\left\{ {\left[ {\Delta {\text{A34}}0\left( {{\text{sample}}} \right) \, - \, \Delta {\text{A34}}0 \, \left( {{\text{blank}}} \right)} \right]/{\text{t}}} \right\}/\left( {\varepsilon *{\text{L}}} \right)$$

### Measurement of RNS scavenging activity of MoSe_2_-PVP NPs

The reactive nitrogen substances (RNS) scavenging activity was evaluated using DPPH· as the probe. DPPH was weighed 1.0 mg and dissolved in 32 mL of anhydrous ethanol. Then, 2 mL of the DPPH solution was mixed with different concentrations of MoSe_2_-PVP NPs solutions (300 µL, 0, 0.05, 0.1, 0.2, and 0.4 mg/mL) and incubated in an oven at 37 °C for 5 min. Moreover, MoSe_2_-PVP NPs with concentration of 0.20 mg/mL was cultured at 50 °C for 25 min and then added thereinto and cultured at 37 °C for 5 min. The resultant solution was scanned via a UV–Vis spectrophotometer in the wavelength range of 410–750 nm (Shimadzu, Japan) and the kinetic changes of solution absorbance with time at 519 nm were also examined. And its scavenging ratio was calculated as Eq. 4:

4 $${\text{Scavenging ratio }}\left( {\text{\% }} \right){ = }\frac{{{\text{A}}_{{0}} {\text{ - A}}_{{1}} }}{{{\text{A}}_{{0}} }}{\text{ * 100\% }}$$

where A_0_ is the absorbance of the blank (DPPH + ethanol), A_1_ is the absorption of the experimental group (DPPH + ethanol + MoSe_2_-PVP NPs).

### In vitro cytocompatibility

Mouse fibroblasts (L929) and acinar pancreatic cell line (266–6) were purchased from the cell bank of the Chinese Academy of Sciences. The cytocompatibility of MoSe_2_-PVP NPs was assessed in vitro by means of CCK-8. L929 and 266–6 cells were cultured in a 96-well tissue culture plate overnight at 37 °C. Each well was filled with different concentration of MoSe_2_-PVP NPs (0–0.1 mg/mL, dispersed in the RMPI1640). After 48 h, we replaced the old solution with fresh RMPI1640 containing CCK-8 test solution and incubated the plate for 2 h. The absorbance of each well at 450 nm was read using a micro-reader (Victor 3) to calculate the cell viability. Subsequently, each well was washed with PBS, and an inverted phase contrast microscope (Leica DM IL, Germany) was used to observe the cell survival and death outcomes.

### In vitro hemocompatibility

Mouse red blood cells (mRBCs) were prepared by centrifuging 1 mL of mouse whole blood at 5000 rpm for 3 min and washed 3 times with PBS [41, 46]. After dilution, 1.2 mL of deionized water and 1.2 mL of PBS solution were set up as positive control and negative control respectively, and the same volume of MoSe_2_-PVP NPs with different concentrations were used as experimental groups. Then, 0.4 mL of mRBCs were added to the control and experimental groups and incubated for 2 h. Next, the absorbance value of the supernatant was measured by centrifugation, and the hemolysis rate (HR) was calculated according to the formula (5):

5 $${\text{HR }}\left( {\text{\% }} \right){\text{ = (A - B)/(B - C) * 100\% }}$$

A: the absorbance of the experimental groups; B: absorbance of the negative control groups; C: absorbance of the positive control groups [47].

### In vivo animal tissue safety evaluation and biodistribution

All animal experiments were carried out in accordance with the protocols approved by the Experimental Animal Center of Changhai Hospital of the Second Military Medical University and the policies of the Ministry of Health. In brief, Kunming mice (from Shanghai Laboratory Animal Center) of similar weight were divided into control and experimental groups (n = 3) and the experimental groups was injected with 200 µL of 1 mg/mL MoSe_2_-PVP NPs solution through the tail vein, while the control group was injected with an equal volume of saline. The mice were fed freely and their weight changes were recorded. Next, mice were euthanised after 1, 7, 14 and 28 days respectively, serum biochemical parameters and blood routine were obtained by collecting mouse eyeball blood. The heart, liver, spleen, lung and kidney tissues of the mice were dissected and partly stained with H&E to assess the effect of MoSe_2_-PVP on tissue safety. Moreover, the main organs were ablated in aqua regia to quantify the biodistribution of Mo ions by ICP-OES (Agilent 700, Series Agilent Technologies).

### Scavenging ROS ability of MoSe_2_-PVP NPs in vitro

RAW264.7 cells were inoculated in 6-well cell culture plates at a density of 100,000 cells/well overnight, and the cells were divided into the control group, H_2_O_2_ stimulation group and MoSe_2_-PVP NPs treatment group. Firstly, the cells were incubated with MoSe_2_-PVP NPs (0.2 mg/mL) for 2 h and then induced inflammation with H_2_O_2_ (500 µM) for 24 h. After incubation as described above, cells were gently rinsed 3 times with serum-free medium to remove free MoSe_2_-PVP NPs. Then, after incubation with 2,7-dichlorofluorescein diacetate (DCFH-DA, 30 min), flow cytometry (BD, FACSCalibur) was used to quantify intracellular ROS levels in each group, respectively, and quantified by FlowJo X 10.0.7 software.

Furthermore, the RAW264.7 cells were inoculated into 6-well plates and cultured. Then, DMEM containing MoSe_2_-PVP NPs dispersion (0.2 mg/mL) was further incubated with the cells for 4 h. Next, cells were treated with 500 µM H_2_O_2_ for 24 h. Finally, cells were stained with propidium iodide/Annexin V-FITC and fluorescence was recorded by flow cytometry (BD, FACSCalibur). Finally, using 96-well culture plates, RAW264.7 cells were incubated with MoSe_2_-PVP NPs (0, 50, 100, and 200 μg/mL) for 4 h at 37 °C. Then, after treatment with H_2_O_2_ (500 μM) at 37 °C for 24 h, cell viability was measured using the CCK-8 method.

### Effectiveness of MoSe_2_-PVP NPs for pancreatitis in vivo

The animal model of acute pancreatitis were established on C57BL/6 mice to assess the therapeutic efficiency of MoSe_2_-PVP NPs. Mice were grouped as follows (n = 3): control group, positive group, experimental group. The mice were fasted for 12 h at the beginning of the experiment, and the positive group and MoSe_2_-PVP group were given intraperitoneal injection of caerulein (0.2 mL per hour, 12 doses). Next, mice in the experimental group were injected with MoSe_2_-PVP (0.2 mL in PBS, 1 mg/mL) after the 1^st^ and 8^th^ injection of caerulein. After 24 h, mice were euthanized and the blood was collected for assays. Pancreatic tissue specimens were taken and fixed in paraformaldehyde and assessed for pathology examination by hematoxylin eosin (H&E) staining. Moreover, we explored the enrichment of MoSe_2_-PVP on pancreatic sites by Micro-CT (Venus001) and ICP. Firstly, C57 black mice were divided into a control group and two experimental groups (n = 3). The normal group was injected with 200 μL PBS, and the experimental groups were intravenously injected with equal amounts of MoSe_2_-PVP NPs (1 mg/mL in PBS). Then, normal group and experimental groups mice (at different time points after the materials injection: 12 h, 24 h) were euthanized, and a portion of the pancreatic tissue was removed and fixed in paraformaldehyde for Micro-CT scanning, and the other part was ablated with aqua regia for ICP analysis.

### Statistical analysis

Unless special indicated, sample size per group is 3 and the above figures are expressed as mean ± standard deviation (mean ± SD). Multiple statistical comparisons of experimental data were performed using one-way ANOVA using the ORIGIN software platform. If p < 0.05 is indicated by *, if p < 0.01 is indicated by **, if p < 0.001 is indicated by ***.

## Results and discussion

### Design and characterization of MoSe_2_-PVP NPs

**Scheme 1:**
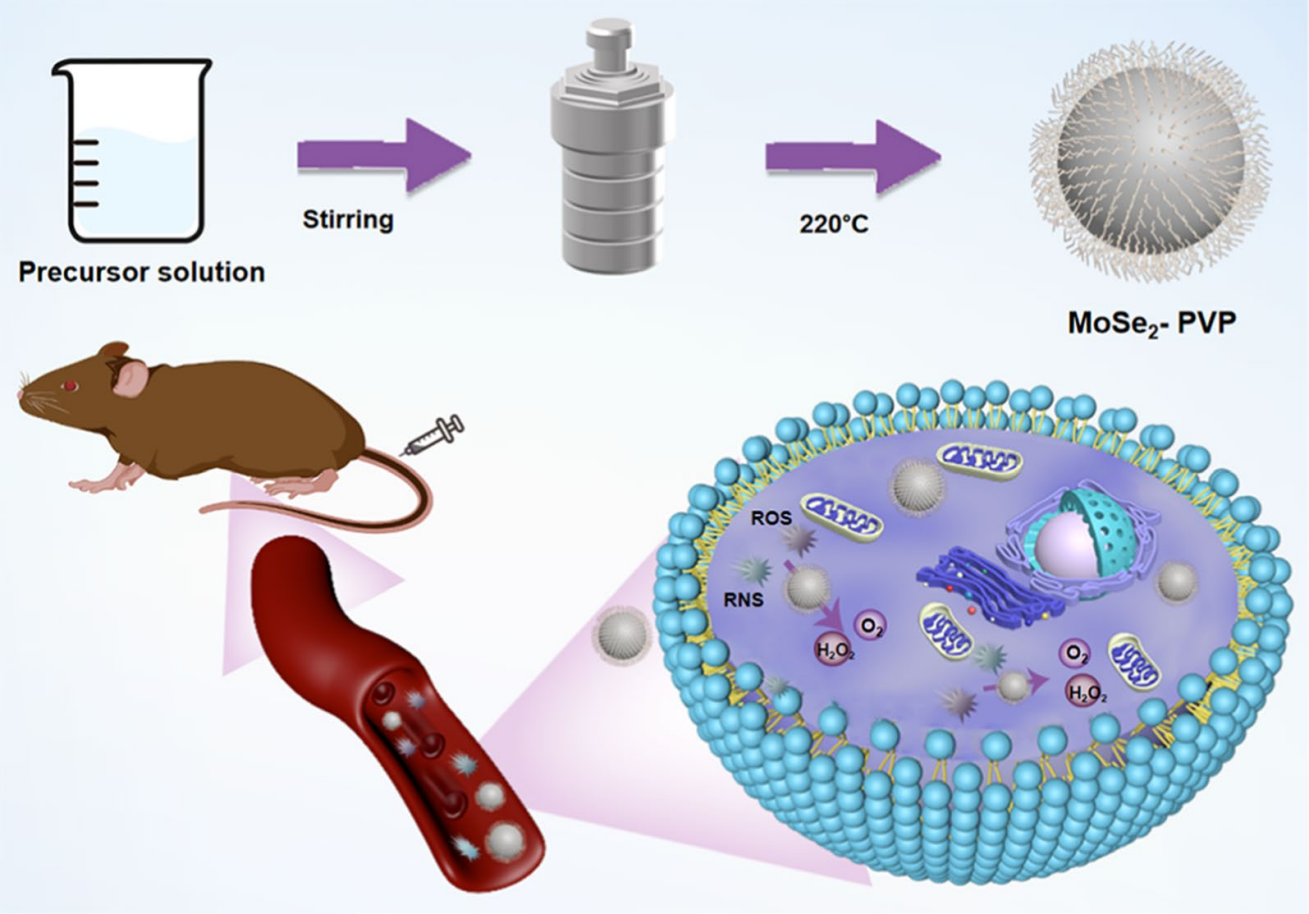
Schematic diagram of the synthesis steps of MoSe_2_-PVP NPs and the therapeutic mechanism for alleviating the acute pancreatitis by scavenging RONS

**Fig. 1:**
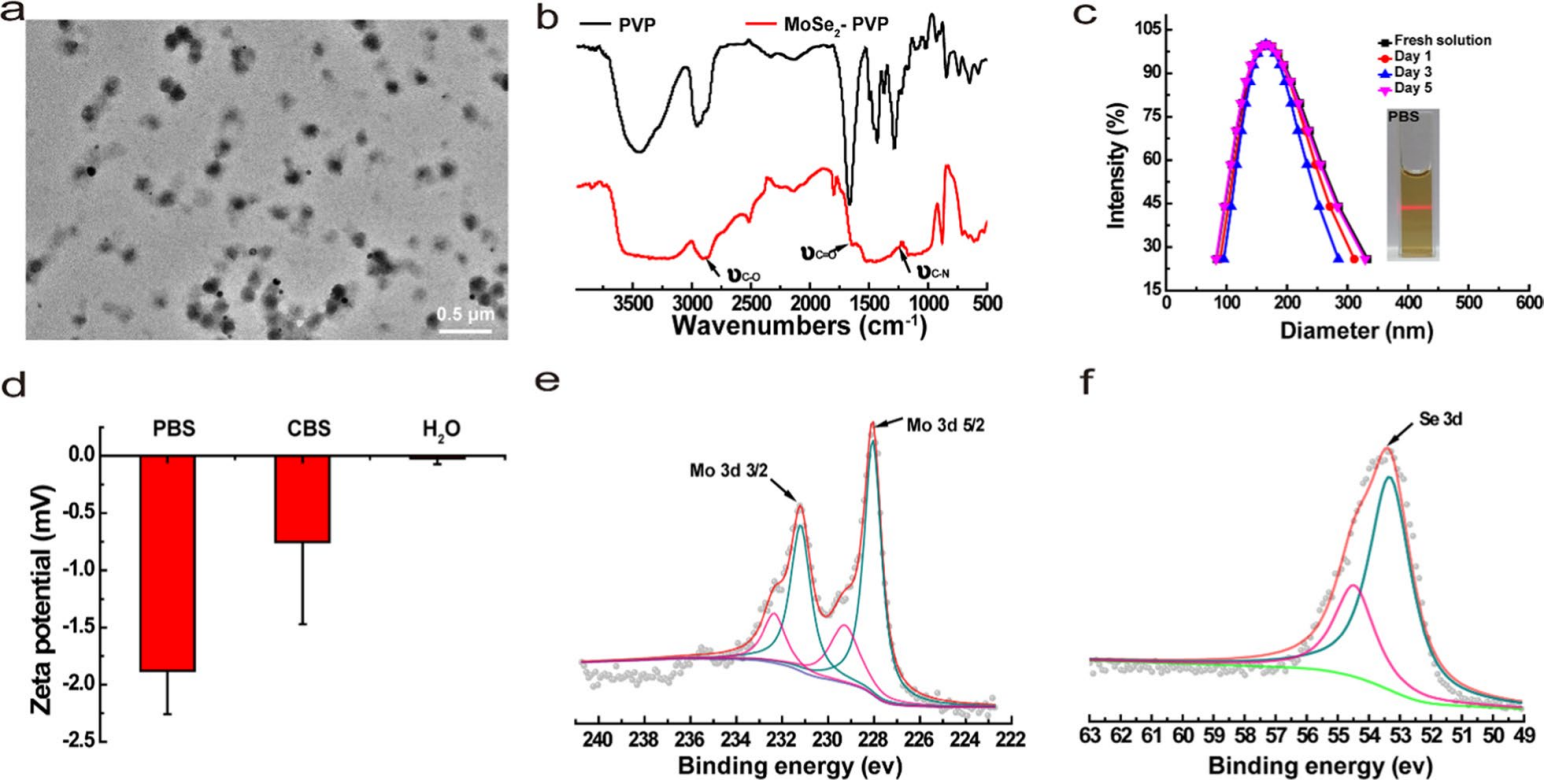
Characterization of MoSe_2_-PVP NPs. **a** TEM images of MoSe_2_-PVP NPs. **b** FTIR spectra of PVP and MoSe_2_-PVP NPs. **c** Dynamic light scattering and Tyndall photographs of MoSe_2_-PVP NPs in PBS. **d** Zeta potentials of MoSe_2_-PVP NPs in PBS, CBS, and H_2_O. XPS spectra of **e** Mo 3d, and **f** Se 3d orbits for the MoSe_2_-PVP NPs

In this study, we successfully synthesized biodegradable MoSe_2_-PVP NPs from a mixture of sodium molybdate dehydrate, L-selenocysteine, and PVP by a one-pot hydrothermal method (Scheme 1). During the hydrothermal process, Mo atoms reacted with Se atoms, and PVP was simultaneously modified on the material surface due to the chelate coordinating effect of O and Mo atoms. The structure and morphology of the final product observed by transmission electron microscopy (TEM) and scanning electron microscopy (SEM) revealed a distinct nanoparticle structure with an average particle diameter of 119.39 ± 13.94 nm (Fig. 1a, Additional file 1: Fig. S1a, and S1b). FT-IR studies confirmed that PVP was successfully modified on the MoSe_2_-PVP NP surface. Compared with the standard PVP spectrum, the peak caused by the vibration of carbonyl groups is located at approximately 1625 cm^−1^, and the C-N stretching vibrational band is centered at 1241 cm^−1^. The strong peak at 2895 cm^−1^ corresponds to the C-O stretching vibration of PVP (Fig. 1b). In addition, no significant differences between the sizes of MoSe_2_-PVP NPs determined in water, saline, PBS and at different time points were observed. The average DLS size was approximately 200 nm, and the NP solutions exhibited an apparent Tyndall effect under light irradiation (Fig. 1c and Additional file 1: Fig. S1b-d), indicating excellent colloidal stability of the synthesized MoSe_2_-PVP NPs. The TEM and DLS particle sizes were slightly different because the dimensions measured by DLS represented hydration kinetic diameters with a certain degree of hydration. Meanwhile, the negative ζ-potentials of PBS, CBS and H_2_O also contributed to the high colloidal stability of MoSe_2_-PVP NPs (Fig. 1d). The chemical composition of the obtained product was determined by XPS (Figs. 1e and f), and the peaks with binding energies of 230.5, 227.4, and 53.4 eV corresponded to Mo 3d_3/2_, Mo 3d_5/2_, and Se 3d species, respectively, which indicated that the product chemical formula was MoSe_2_.

### Biodegradability of MoSe_2_-PVP NPs

**Fig. 2:**
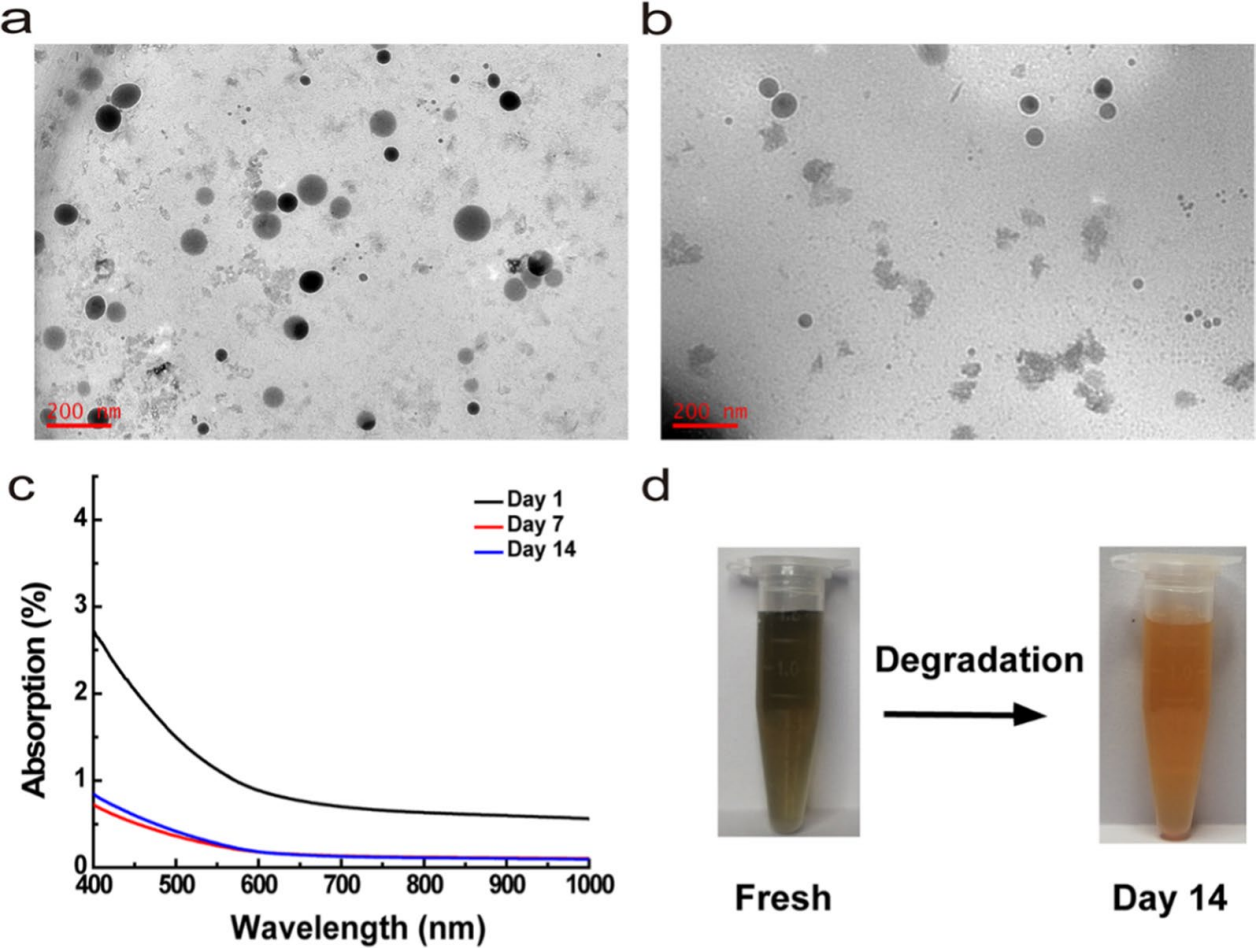
The TEM images of MoSe_2_-PVP NPs degradation process of **a** fresh solution and **b** day 14. **c** UV–vis-NIR absorption spectrum of MoSe_2_-PVP NPs degradation process, and **d** corresponding photographs

Although different types of nanoparticles were studied as artificial nanoenzymes with high total antioxidant capacities, their poor biodegradation properties remained a considerable challenge. Herein, we found that the one-pot synthesized MoSe_2_-PVP NPs were biodegradable. Morphological changes of the prepared samples were observed during the degradation of MoSe_2_-PVP by TEM (Fig. 2a, b). In PBS, the freshly prepared MoSe_2_-PVP NPs possessed a uniform nanoparticle morphology; however, the typical NP structure gradually collapsed and eventually disappeared with increasing storage time. The degradation process was monitored by a UV–vis spectrometer. The obtained spectra revealed that the material absorbance gradually decreased over time at high degradation rate within the first 7 d and approached 0 after 14 d (Fig. 2c). Photographs of the degraded MoSe_2_-PVP NPs in PBS (1.5 mL centrifuge tubes) were recorded, and the color fading of their solution confirmed the material degradation (Fig. 2d). This phenomenon was likely caused by the oxidization of MoSe_2_-PVP NPs to selenite, and the degraded MoSe_2_-PVP NPs was transformed into a precipitate; however, its specific reasons need to be explored in future studies. It is worth noting that the DLS size of the MoSe_2_-PVP NPs did not change significantly at the initial degradation stage (Fig. 1c). This may be because that the undegraded particles still maintained their good colloidal stability in the solution.

### Antioxidant capacity measurements

**Fig. 3:**
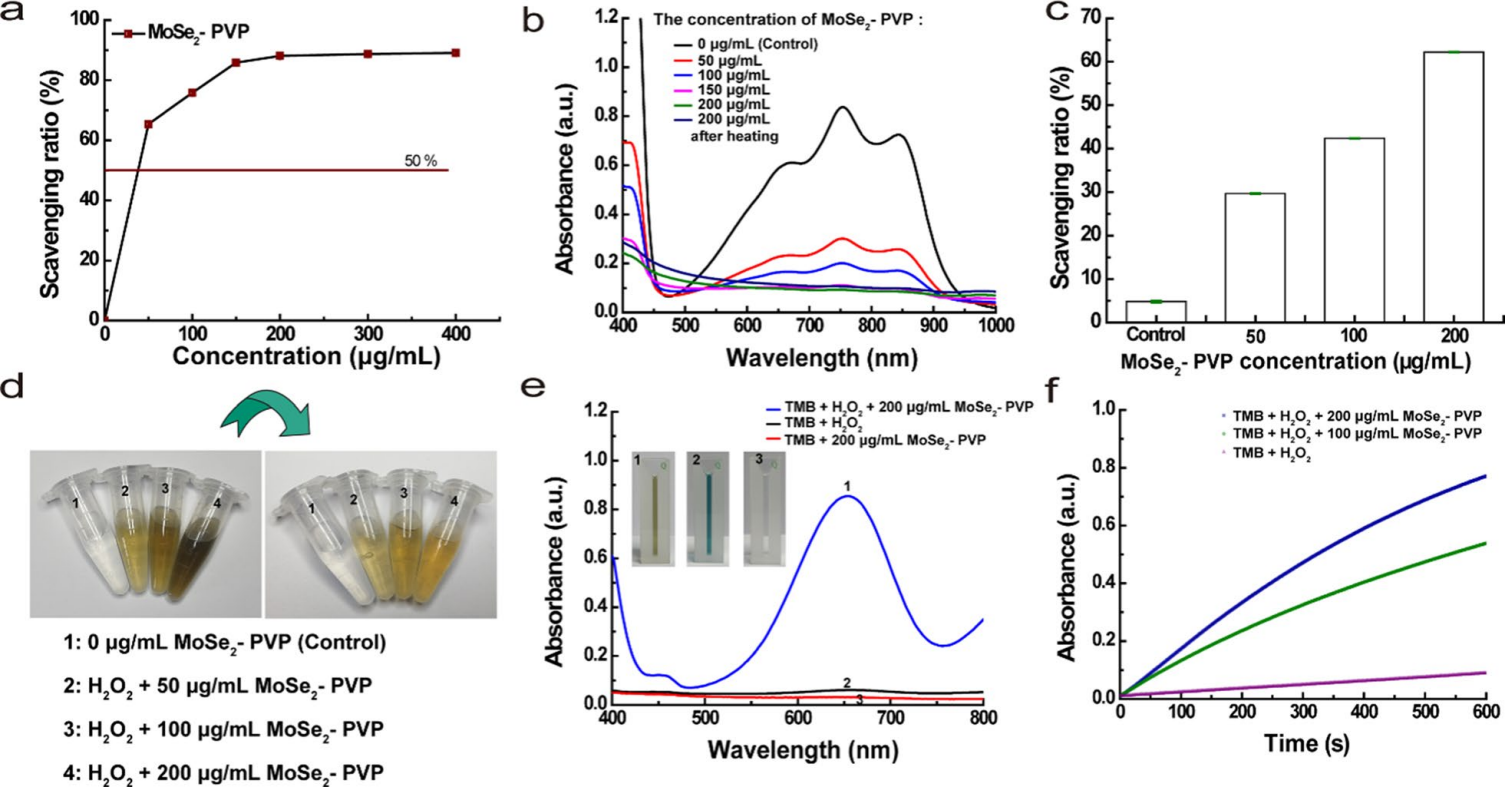
**a** The ABTS^+^ scavenging ratio of MoSe_2_-PVP NPs. **b** The UV–Vis absorption spectra of ABTS^+^ solution after treated with different concentrations of MoSe_2_-PVP NPs. **c** The scavenging ratio of MoSe_2_-PVP NPs, and **d** the photographs of before and after reaction. **e** The UV–Vis absorption spectra of MoSe_2_-PVP NPs for TMB coloration under the presence of H_2_O_2_, and **f** the kinetic change at 650 nm of MoSe_2_-PVP NPs for TMB coloration under the presence of H_2_O_2_

The total antioxidant capacity of MoSe_2_-PVP NPs was measured by the ABTS radical method. After adding MoSe_2_-PVP NPs to the ABTS^+^ solution, its characteristic green color faded immediately, and the absorbance of ABTS^+^ at 752 nm decreased. Moreover, a larger absorbance decrease was observed with increasing concentration of MoSe_2_-PVP NPs, and approximately half of the ABTS^+^ radicals were removed at low NP concentrations (below 50 μg/mL; Fig. 3a). These data indicate that MoSe_2_-PVP NPs can potentially serve as artificial nanoenzymes and possess high antioxidant capacity. Moreover, natural enzymes are temperature-sensitive, and their structure is easily damaged by hyperthermia. Accordingly, the thermal stability of MoSe_2_-PVP NPs was investigated by continuously heating them in a water bath to 50 °C for 25 min and immediately measuring the overall antioxidant capacity. As shown in Fig. 3b and Additional file 1: Fig. S2a, the absorbance decrease and color fading degree of the MoSe_2_-PVP NP solution (200 μg/mL) did not change after heating to 50 °C for 25 min, suggesting that the antioxidant capacity of MoSe_2_-PVP NPs was thermally stable.

### Scavenging effect of MoSe_2_-PVP NPs on H_2_O_2_

CAT is an indispensable enzyme in the biological defense system because it can remove excessive H_2_O_2_ species from the body and protect cells from their toxic effects. In the presence of CAT or CAT simulants, H_2_O_2_ is catalytically decomposed into oxygen and water via the reaction 2H_2_O_2_ = O_2_↑ + 2H_2_O. In this section, we evaluated the CAT mimetic activity of MoSe_2_-PVP NPs by monitoring the H_2_O_2_ degradation process. The characteristic absorption of H_2_O_2_ at 240 nm decreased from 0.58 to 0.25 after the addition of MoSe_2_-PVP NPs (0.2 mg/mL), while the absorbance of the control remained almost unchanged (Additional file 1: Fig. S3a). The H_2_O_2_ scavenging ratio was then calculated. As shown in Fig. 3c, the H_2_O_2_ scavenging ratio of the control group was less than 10%, while that of the experimental group exceeded 60% at an NP concentration of 0.2 mg/mL, indicating that MoSe_2_-PVP NPs could efficiently catalyze the degradation of H_2_O_2_. Moreover, the color fading of the MoSe_2_-PVP NP solution after the reaction (Fig. 3d) also confirmed the scavenging effect of MoSe_2_-PVP NPs on H_2_O_2_.

### POD-like behavior of MoSe_2_-PVP NPs

POD is an oxidoreductase enzyme that oxidizes benzidine to its blue-colored derivative compound. In this section, we examined the POD-like behavior of MoSe_2_-PVP NPs using a TMB substrate (Fig. 3e). The TMB oxidation peak gradually increased at 650 nm; however, in the absence of H_2_O_2_ or MoSe_2_-PVP NPs, the absorption of TMB remained almost unchanged, which confirmed the POD mimetic activity of MoSe_2_-PVP NPs. Furthermore, the kinetic change at 650 nm also reflected POD activity (Fig. 3f). When MoSe_2_-PVP NPs were present in the reaction system, the absorbance increased steeply and positively correlated with their concentration.

### Scavenging effect of MoSe_2_-PVP NPs on·OH

**Fig. 4:**
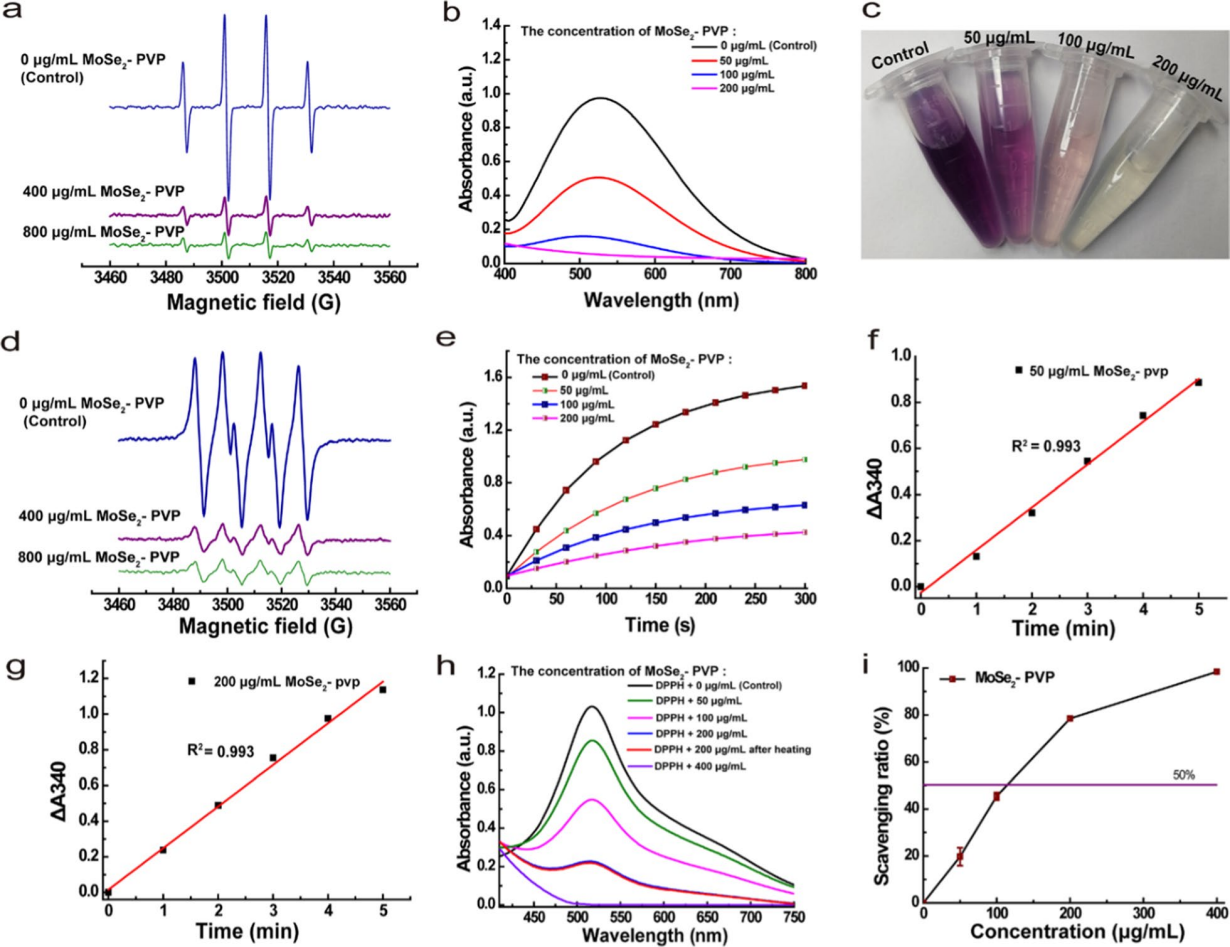
**a** The ESR spectra of DMPO/MoSe_2_-PVP NPs to illustrate the ·OH scavenging ability of MoSe_2_-PVP NPs. **b** The UV–vis absorption spectra of MoSe_2_-PVP NPs incubated with salicylic acid and **c** corresponding photographs of **b**. **d** The ESR spectra of DMPO that incubated with MoSe_2_-PVP NPs to illustrate the ·O^2−^ scavenging of MoSe_2_-PVP NPs. **e** The scavenging ratio of DPPH· for MoSe_2_-PVP NPs. The GPx-like activity of MoSe_2_-PVP NPs at **f** 50 μg/mL and at **g** 200 μg/mL. **h** The UV–vis absorption spectra of DPPH· solution after incubated with MoSe_2_-PVP NPs. **i** The scavenging ratio of MoSe_2_-PVP NPs for DPPH

The ·OH scavenging properties of MoSe_2_-PVP NPs were examined by paramagnetic resonance spectroscopy (ESR). When captured by DMPO, ·OH produces a typical ·OH ESR peak. After the addition of MoSe_2_-PVP NPs to the reaction system, the ·OH peak significantly decreased with an increase in the NP concentration (Fig. 4a). The ·OH scavenging properties of MoSe_2_-PVP NPs were further evaluated using SA. The ·OH radical can react with SA to produce purple-colored 2,3-dihydroxybenzoic acid with a specific absorption wavelength of 510 nm. Therefore, the ·OH scavenging effect of the test compound can be evaluated by measuring the absorbance of the reaction system at 510 nm. As shown in Fig. 4b and c, SA was easily oxidized by ·OH to produce a purple solution without MoSe_2_-PVP NPs; however, after the MoSe_2_-PVP NPs addition, the characteristic peak at 510 nm ultimately disappeared, and the color of the reaction solution gradually faded in accordance with the ESR results, further confirming the high ·OH scavenging activity of MoSe_2_-PVP NPs.

### Scavenging effect of MoSe_2_-PVP NPs on superoxide anions radicals

SOD, an important antioxidant enzyme in living organisms, can catalyze the dismutation of superoxide anions to produce H_2_O_2_ and O_2_ species, thereby preventing oxidative stress-induced diseases. The autoxidation of pyrogallol generates ·O^2−^ radicals, therefore, the SOD-like properties of a sample can be examined by measuring its inhibition rate of the autoxidation of pyrogallol. The SOD-like activity of MoSe_2_-PVP NPs was first studied by spin capture technology using DMPO to capture free radicals. As shown in Fig. 4d, the control group (without MoSe_2_-PVP NPs) generated a classical six-fold superoxide radical peak whose intensity rapidly decreased with the addition of MoSe_2_-PVP NPs, indicating an efficient concentration-dependent elimination of O^2−^ species. The SOD-like properties of MoSe_2_-PVP NPs were further assessed using a pyrogallol method. Typically, pyrogallol is prone to autoxidation under alkaline conditions and produces a yellow-colored compound, which can be monitored by UV colorimetry at a characteristic absorbance wavelength of 320 nm. Thus, pyrogallol was employed to assess the SOD-like properties of MoSe_2_-PVP NPs. As shown in Fig. 4e, under the utilized experimental conditions, the light absorbance of pyrogallol changed to 1.4 due to oxidation. In contrast, after adding various concentrations of MoSe_2_-PVP NPs to the reaction solution, the corresponding absorbance values gradually decreased at the same time point. For example, the absorbance of approximately 0.3 was achieved at a NP concentration of 0.4 mg/mL, further confirming the high superoxide anion radical scavenging activity of MoSe_2_-PVP NPs.

### GPx-like activity of MoSe_2_-PVP NPs

GPx plays a key role in maintaining the in vivo H_2_O_2_ levels because it can recruit glutathione (GSH) to catalyze H_2_O_2_ and organic peroxides to produce water or organic alcohols. In this study, the GPx-like characteristics of MoSe_2_-PVP NPs were examined using the total GPx assay kit (NADPH method). The GPx activity was measured at a wavelength of 340 nm by UV colorimetry on an enzyme calibrator using organic peroxide reagent as a substrate, and the final enzyme activity was calculated according to manufacturer’s instructions. The obtained results revealed that the NADPH consumption increased with increasing sample concentration (Additional file 1: Fig. S3c). The enzyme activities of MoSe_2_-PVP NPs calculated at NP concentrations of 50 and 200 μg/mL were 103.317 and 132.415 mU/mL, respectively, which indicated that the MoSe_2_-PVP NPs exhibited a high GPx-like activity (Fig. 4f and g).

### RNS scavenging activity of MoSe_2_-PVP NPs

The RNS scavenging activity of MoSe_2_-PVP NPs was evaluated by measuring the scavenging efficiency of DPPH free radicals (a typical RNS). Ethanolic solutions of DPPH radicals are purple-colored with a characteristic absorption wavelength of 519 nm. After DPPH radicals were removed by MoSe_2_-PVP NPs, their solutions faded, and the degree of discoloration was proportional to the change in absorbance quantified by spectrophotometry. As shown in Fig. 4h and Additional file 1: Fig. S3b, the elimination of DPPH positively correlated with the NP concentration and time, and the DPPH characteristic absorption peak at 519 nm disappeared when the MoSe_2_-PVP NP concentration reached 400 μg/mL. The scavenging ratio was calculated according to Eq. (4), and the obtained results are shown in Fig. 4i. More than 50% of DPPH· was scavenged at MoSe_2_-PVP NPs concentrations higher than 200 μg/mL, confirming that these NPs were able to mimic multiple enzymatic activities and scavenge free radicals. Moreover, the RNS scavenging activity of MoSe_2_-PVP NPs was thermally stable and not significantly affected by heating to 50 °C (Fig. 4h).

### Biocompatibility of MoSe_2_-PVP NPs in vitro

**Fig. 5:**
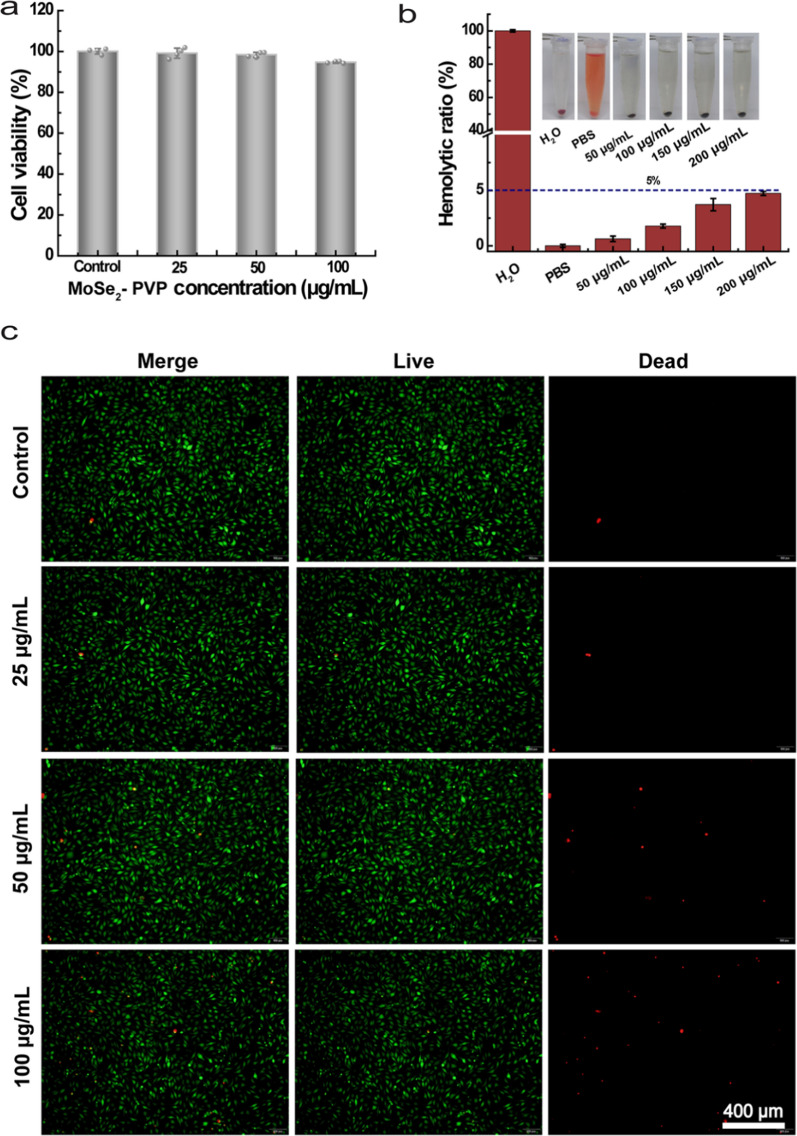
The safety of MoSe_2_-PVP NPs in vitro. **a** Cells viabilities of L929 after cultured with different concentrations of MoSe_2_-PVP NPs. **b** Hemolysis ratio of mRBCs incubated with MoSe_2_-PVP NPs and the photographs of mRBCs after centrifugation in set. **c** The photographs of Live/Dead staining corresponding to **a**. Representative graphics are shown, n = 3 independent experiments

The results discussed in the previous sections encouraged us to validate the feasibility of using MoSe_2_-PVP NPs in biomedical applications. Regarding the biocompatibility in vitro, we initially co-cultured them with L929 and 266–6 cells and then examined the corresponding cellular activity using the CCK-8 assay. The cell viability was barely affected in the experimental group and remained higher than 94% and 96% even after the MoSe_2_-PVP NPs concentration increased to 100 μg/mL (Fig. 5a and Additional file 1: Fig. S4), indicating their non-cytotoxicity. Afterwards, we observed stained cells under a fluorescence microscope and found that viable cells with green fluorescence dominated the field of view in each group, demonstrating the cell safety of MoSe_2_-PVP NPs (Fig. 5c and Additional file 1: Fig. S4). Moreover, to study the hemocompatibility, different concentrations of MoSe_2_-PVP NPs were incubated with diluted mouse blood for 2 h, and the supernatant was taken to detect the absorbance. The obtained results revealed that erythrocytes in the distilled water group ruptured and that the hemolysis rate was almost 100%, while no rupture or swelling of erythrocytes occurred after the addition of PBS and different concentrations of MoSe_2_-PVP NPs. The measured HP value was less than 5%, indicating that MoSe_2_-PVP NPs caused no damage to erythrocytes (Fig. 5b).

### In vivo histocompatibility evaluation

**Fig. 6:**
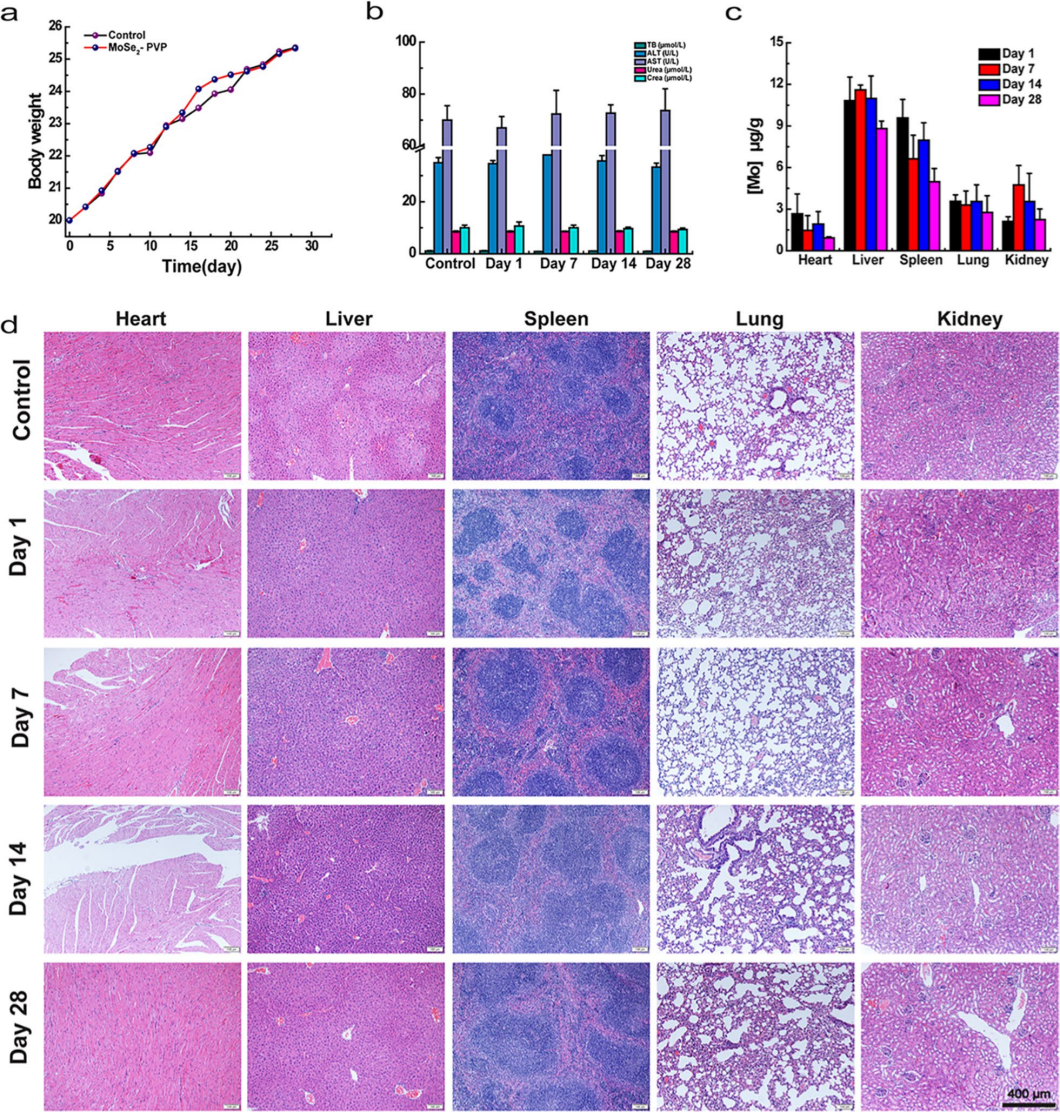
The safety of MoSe_2_-PVP NPs in vivo. **a** KM mice body weight treated with saline or MoSe_2_-PVP NPs (1 mg/mL). **b** The blood biochemistry parameters. **c** Biodistribution of MoSe_2_-PVP NPs in the major organs after intravenous injection for different days. **d** The vivo toxicity evaluation for the major organs of KM mice by H&E staining. Representative graphics are shown, n = 3 independent experiments

The in vivo histocompatibility of MoSe_2_-PVP NPs was monitored by assessing their effects on the blood biochemistry and histopathology of major organs of mice. First, the weight changes of the experimental mice during the entire treatment period were normal and exhibited no significant differences from the values obtained for the healthy mice (Fig. 6a). The biochemical analysis of the serum from the experimental group indicated that its concentrations of the serum liver function indicators (AST and ALT) and kidney function indicators (BUN and CRE) were comparable to those of the control group (Fig. 6b). In addition, the main blood characteristics (including WBC, RBC, HB, HCT, MCV, MCH, MCHC, RDW, and PLT) of the control group were consistent with the values obtained for the experimental group (Additional file 1: Fig. S5a–i). Meanwhile, we explored the biodistribution of MoSe_2_-PVP NPs in the major organs of mice by ICP-AES. As shown in Fig. 6c, the liver exhibited the highest dose distribution of MoSe_2_-PVP NPs followed by the spleen, while NPs were less distributed in the heart, kidney, and lung. Moreover, the accumulation of MoSe_2_-PVP NPs in these organs gradually decreased with the feeding duration. Further pathological sections (Fig. 6d) showed the absence of apparent inflammation or tissue damage in the heart, liver, spleen, lung, or kidney after the MoSe_2_-PVP NPs injection with the subsequent feeding for 1, 7, 14, and 28 d. These findings indicate that MoSe_2_-PVP NPs exhibit high histocompatibility.

### Protecting cells from ROS in vitro

**Fig. 7:**
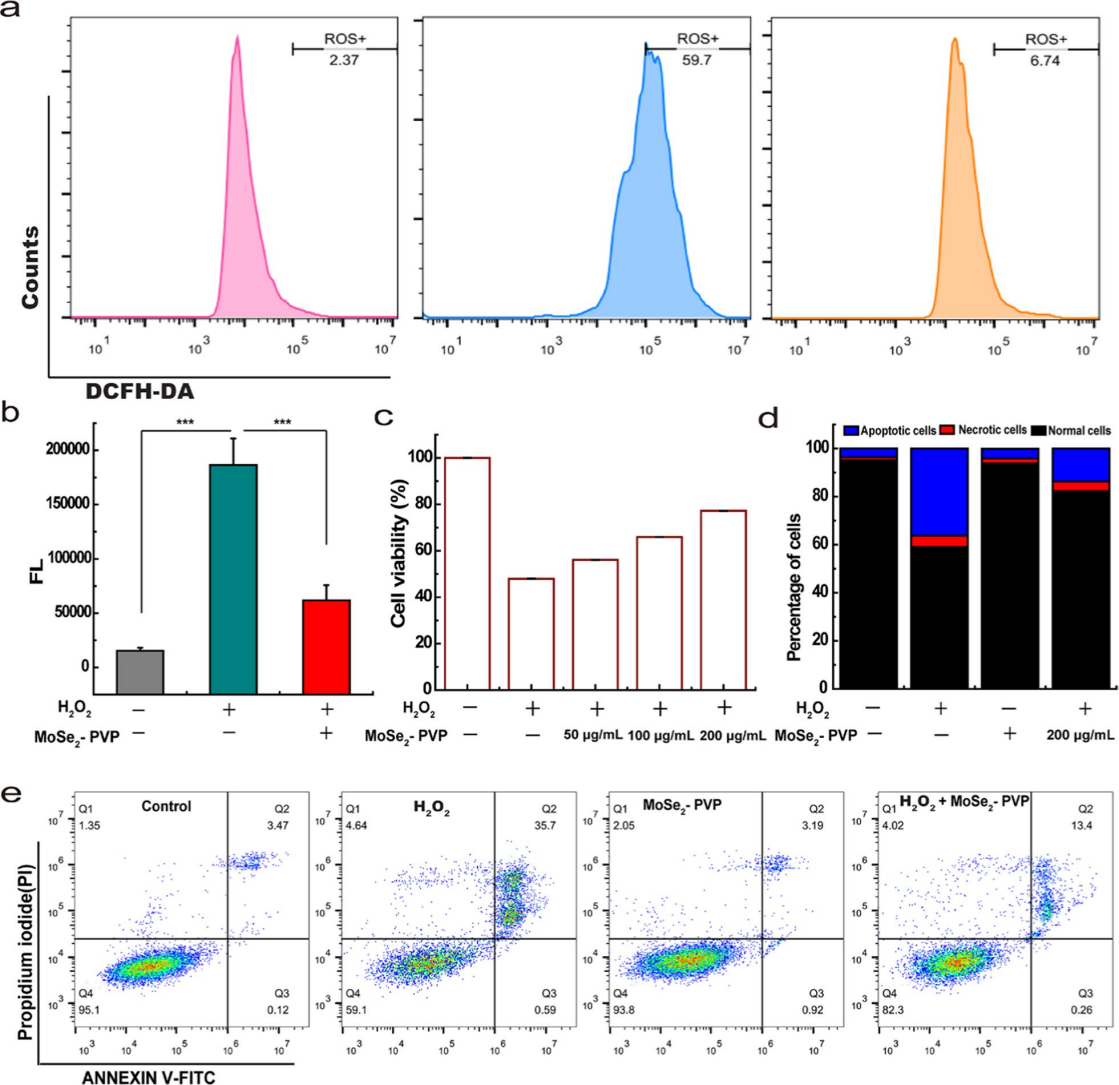
Cytoprotection effect of MoSe_2_-PVP NPs in vitro. **a** Quantitative analysis of ROS levels in RAW264.7 cells (left: healthy cells; middle: H_2_O_2_-treated RAW264.7 cells; right: H_2_O_2_/MoSe_2_-PVP NPs-treated 264.7 cells). **b** Quantitative analysis of ROS levels in RAW264.7 cells. **c** Viability of RAW264.7 cells after treatment with 500 µM H_2_O_2_ and different concentrations of MoSe_2_-PVP NPs (H_2_O_2_ -: without the H_2_O_2_; H_2_O_2_ + : with the H_2_O_2_. MoS_2_-PVP -: without the MoS_2_-PVP). A histogram diagram (**d**) and a scatter diagram (**e**) of cell apoptosis and necrosis distribution in untreated and MoSe_2_-PVP NPs-treated RAW264.7 cells. Representative graphics are shown, n = 3 independent experiments

Owing to the superior anti-oxidizing properties and biocompatibility of MoSe_2_-PVP NPs, we investigated their ability to remove overproduced intracellular oxidizing species by treating the RAW264.7 cell line model with H_2_O_2_. DCFH-DA, an oxidation-sensitive fluorescent dye, was used as a probe to label ROS, and intracellular ROS levels were determined by flow cytometry. Compared with the intracellular ROS level in the H_2_O_2_-treated RAW264.7 cells, its magnitude decreased from 59.7 to 6.74 counts after the addition of MoSe_2_-PVP NPs (Fig. 7a, b, ***p < 0.001), which was comparable to the value obtained for the positive control (healthy cells, Fig. 7b). These results clearly demonstrate that MoSe_2_-PVP NPs can effectively inhibit the overproduction of ROS in H_2_O_2_-treated cells, which leads to the subsequent alleviation of AP in mice. Furthermore, the CCK-8 data visually confirmed that the excessive H_2_O_2_ could induce cell death, while MoSe_2_-PVP NPs exhibited a concentration-dependent protective effect on cells by scavenging ROS. The survival rates of the H_2_O_2_-treated cells determined at MoSe_2_-PVP NPs concentrations of 0.05, 0.1, and 0.2 mg/mL were 56%, 66%, and 77%, respectively (Fig. 7c). The observed cell-protective efficiency was further confirmed by flow cytometry. As shown in Fig. 7d, e, approximately 36.29% of the gated cells were damaged by H_2_O_2_; however, this value decreased to 13.66% after the addition of MoSe_2_-PVP NPs. It is noteworthy that MoSe_2_-PVP NPs alone damaged only 4.11% of all cells, which further confirmed the cytocompatibility of the artificial nanoenzyme.

### In vivo therapeutic efficacy for AP mice

**Fig. 8:**
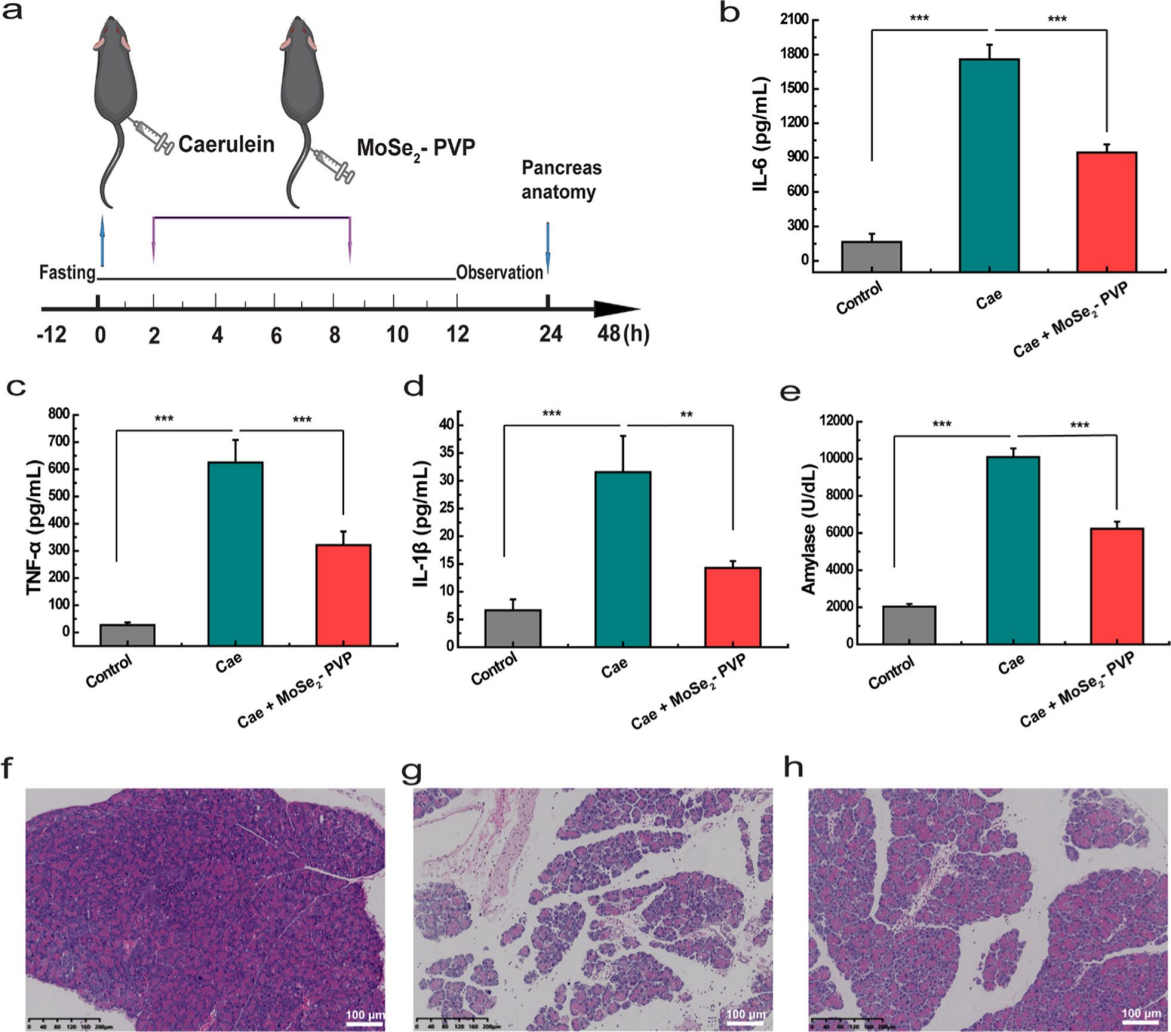
Anti-inflammatory treatment of AP in vivo. **a** Schematic diagram of the establishment and treatment protocol of AP mice model. The levels of **b** amylase, **c** IL-6, **d** TNF-α, and **e** IL-1β from various groups. **f**–**h** H&E staining of pancreatic tissue from various groups (**f**: healthy mice; **g**: caerulein-induced AP mouse model; **h**: caerulein-induced AP mouse that received the MoSe_2_-PVP NPs injection)

Owing to the high antioxidant activity and biocompatibility of MoSe_2_-PVP NPs, we investigated their therapeutic effect on a mouse model of the caerulein-induced AP via tail vein injection of MoSe_2_-PVP NPs. A schematic diagram of the caerulein-induced AP animal model and material injection process is shown in Fig. 8a. We measured the concentrations of serum amylase, a typical indicator of the pancreatic function, and the inflammatory factors IL-6, IL-1β, and TNF-α. As expected, the serum amylase levels and the expression of inflammatory factors significantly increased (Fig. 8b–e, Cae versus control: *** p < 0.001; Cae versus Cae + MoSe_2_-PVP: *** p < 0.001; Cae versus Cae + MoSe_2_-PVP: ** p < 0.01(Fig. 8d)), confirming the successful AP diagnosis. After the injection of MoSe_2_-PVP NPs (1 mg/mL, 200 µL), the average serum amylase levels in the AP mice decreased from 10,097.2 to 6234 U/dL. Correspondingly, the expressions of inflammatory factors after the treatment with MoSe_2_-PVP NPs also decreased to lower levels (IL-6: from 1575.1 to 944.5 pg/mL, Fig. 8b; TNF-α: from 624.9 to 321.1 pg/mL, Fig. 8c; IL-1β: from 31.5 to 14.3 pg/mL, Fig. 8d). The results obtained for the pathological sections of the pancreatic tissue corroborated the ameliorative effect of MoSe_2_-PVP NPs on AP. As shown in Figs. 8f–h, the control group (healthy mice) had a densely arranged pancreas with a normal structure. In contrast, in the caerulein-induced AP mouse model, diffuse and localized edema of the pancreas accompanied by the partial destruction of the gland and widened stroma were detected. Moreover, a small number of neutrophil infiltrations and dilatation of the interstitial blood vessels were also observed (Fig. 8g). Interestingly, in the MoSe_2_-PVP NP-treated group, the pancreatic proliferation, dilation, tissue destruction, and lesions were less pronounced, and a low number of small blood vessels were observed in the stroma, further confirming the AP amelioration properties of the artificial MoSe_2_-PVP NP nanoenzyme.

**Fig. 9:**
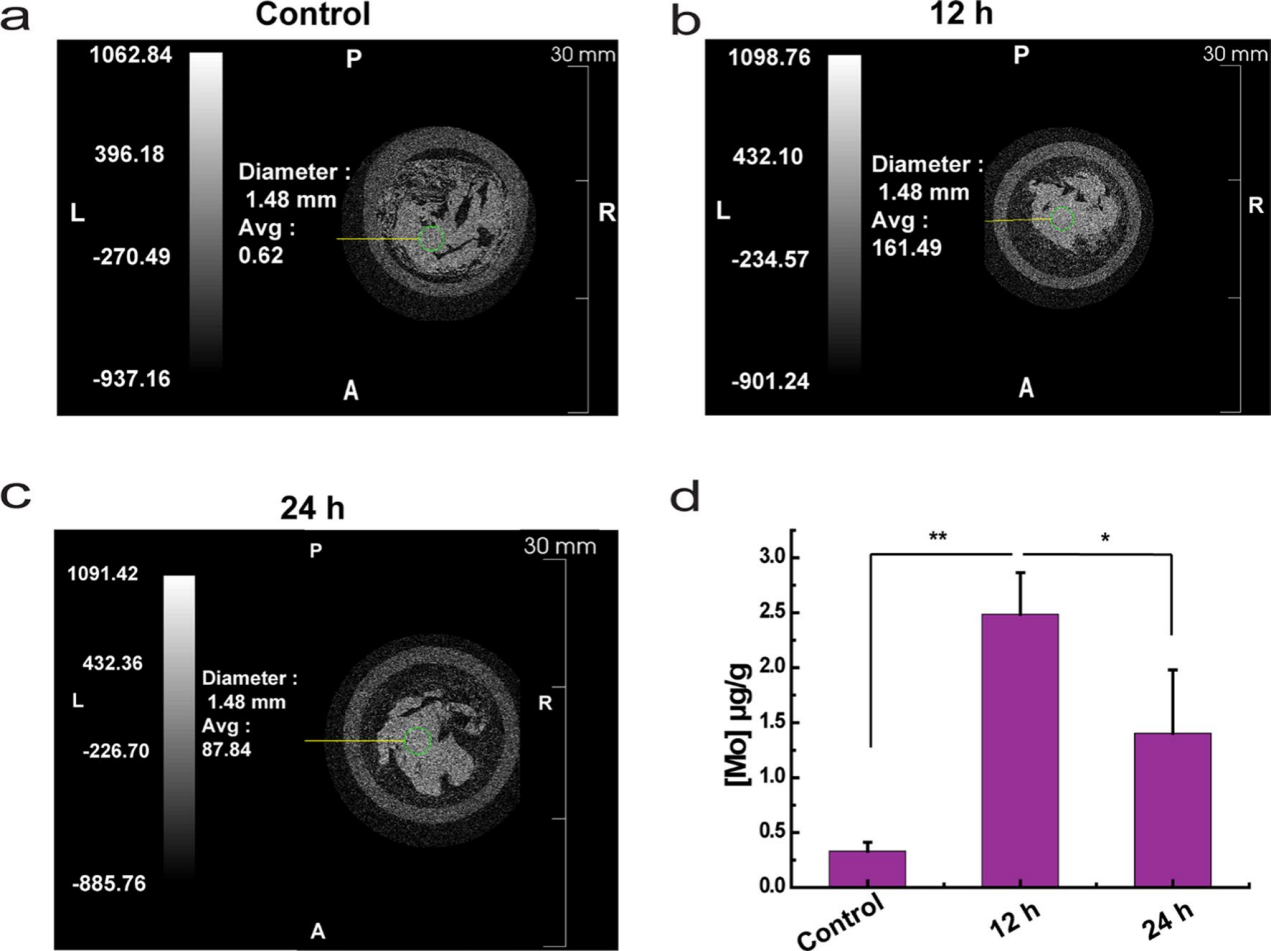
The pancreas CT average values of **a** control, **b** 12 h after intravenous injection, **c** 24 h after intravenous injection. **d** Distribution of Mo ions in the pancreas (** p < 0.01, illustrating the significant Mo accumulation in the pancreas, * p < 0.05, illustrating the significant metabolism of MoSe_2_-PVP NPs in the pancreas)

Furthermore, the results of both Micro-CT scan and ICP indicated an enrichment of MoSe_2_-PVP NPs at the pancreatic site. Compared to the control group, MoSe_2_-PVP NPs accumulated at the pancreatic site after intravenous injection (Fig. 9a–c), and the average CT value (Avg) was 161.49 HU at 12 h. Meanwhile, after the prolonged metabolism in vivo, the accumulation of MoSe_2_-PVP NPs at the pancreatic site decreased, and the Avg CT value decreased to 87.84 HU at 24 h, which was consistent with the ICP data (Fig. 9d). All of the above demonstrated that MoSe_2_-PVP NPs can be concentrated in the pancreatic area and alleviate AP symptoms.

## Conclusion

In this work, we synthesized MoSe_2_-PVP NPs with high physiological stability and biosafety level by a simple inexpensive method, which were able to effectively scavenge mitochondrial and intracellular ROS and RNS for the amelioration of AP. The prepared MoSe_2_-PVP NPs mimicked the intrinsic antioxidant properties of CAT, SOD, POD, and GPx and were able to eliminate a variety of ROS (such as H_2_O_2_, ⋅OH, and ·O^2−^) and RNS (such as DPPH·). Interestingly, the free radical scavenging activity of MoSe_2_-PVP NPs was thermally stable and did not change at high temperatures. Such excellent free radical scavenging properties endowed MoSe_2_-PVP NPs with the ability to protect cells from the ROS-induced damage. Furthermore, MoSe_2_-PVP NPs exhibited high anti-inflammatory efficacy in the caerulein-induced AP mouse model, which significantly inhibited the elevation of the serum amylase level, and reduced the secretion of inflammatory factors in AP-diagnosed mice. In addition, MoSe_2_-PVP NPs were effectively excreted from the mice organs after intravenous injection, indicating high biocompatibility and degradability levels. The findings of this study may open up new avenues for the development of multifunctional nanoenzymes with excellent therapeutic effects.

## Supplementary Information

**Additional file 1: Figure S1**. (a) SEM of MoSe2-PVP NPs. (b) Histogram of diameter distribution of MoSe2-PVP NPs. Dynamic light scattering and Tyndall photographs of MoSe2-PVP NPs in (c) DMEM, (d) saline, and (e) H2O. **Figure S2**. (a) The photographs of scavenging ability of MoSe2-PVP NPs for ABTS+. **Figure S3**. (a) Absorbance of the reaction of H2O2 in the presence of MoSe2-PVP NPs (200 μg/mL) with time; (b) Kinetic change at 650 nm of POD-like of MoSe2-PVP NPs. (c) Kinetic changes at 340 nm of GPx-like of MoSe2-PVP NPs. **Figure S4**. The safety of MoSe2-PVP NPs in vitro. (a) Cells viabilities of 266-6 cells after cultured with different concentrations of MoSe2-PVP NPs. (b) The photographs of Live/Dead staining corresponding to (a), b: control, c: 50 μg/mL, d: 100 μg/mL. Representative graphics are shown, n = 3 independent experiments. **Figure S5**. (a-i) Hematological test results of mice after the injection with MoSe2-PVP NPs.

## Data Availability

All data generated or analyzed during this study are included in this manuscript and its supplementary material.
