# Supplementary material for: Biodegradable MoSe2-polyvinylpyrrolidone nanoparticles with multi-enzyme activity for ameliorating acute pancreatitis
Source: J Nanobiotechnology. 2022 Mar 5;20:113. doi: 10.1186/s12951-022-01288-x (PMC8898412; doi:10.1186/s12951-022-01288-x)
Supplement: Supplementary file 1 — Additional file 1: Figure S1. (a) SEM of MoSe2-PVP NPs. (b) Histogram of diameter distribution of MoSe2-PVP NPs. Dynamic light scattering and Tyndall photographs of MoSe2-PVP NPs in (c) DMEM, (d) saline, and (e) H2O. Figure S2. (a) The photographs of scavenging ability of MoSe2-PVP NPs for ABTS+. Figure S3. (a) Absorbance of the reaction of H2O2 in the presence of MoSe2-PVP NPs (200 μg/mL) with time; (b) Kinetic change at 650 nm of POD-like of MoSe2-PVP NPs. (c) Kinetic changes at 340 nm of GPx-like of MoSe2-PVP NPs. Figure S4. The safety of MoSe2-PVP NPs in vitro. (a) Cells viabilities of 266-6 cells after cultured with different concentrations of MoSe2-PVP NPs. (b) The photographs of Live/Dead staining corresponding to (a), b: control, c: 50 μg/mL, d: 100 μg/mL. Representative graphics are shown, n = 3 independent experiments. Figure S5. (a-i) Hematological test results of mice after the injection with MoSe2-PVP NPs. [file 12951_2022_1288_MOESM1_ESM.docx]

Supporting Information

**Biodegradable MoSe_2_-PVP with multi-enzyme activity ameliorates acute pancreatitis**

Pei Xie,^a,1^ Liying Zhang,^b,1^ Hui Shen,^a,1^ Hang Wu,^a^ Jiulong Zhao,^a,^* Shige Wang, ^b^ and Lianghao Hu ^a,^*


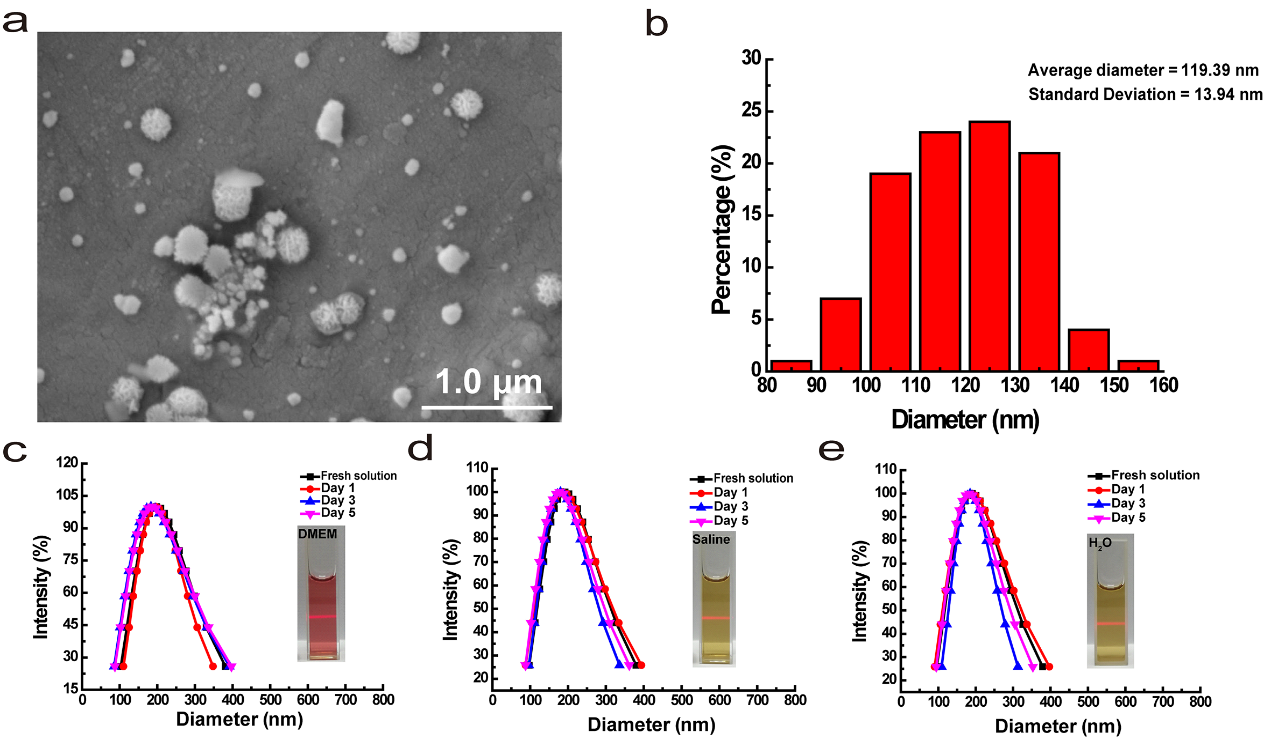


**Figure S1.** (a) SEM of MoSe_2_-PVP NPs. (b) Histogram of diameter distribution of MoSe_2_-PVP NPs. Dynamic light scattering and Tyndall photographs of MoSe_2_-PVP NPs in (c) DMEM, (d) saline, and (e) H_2_O.


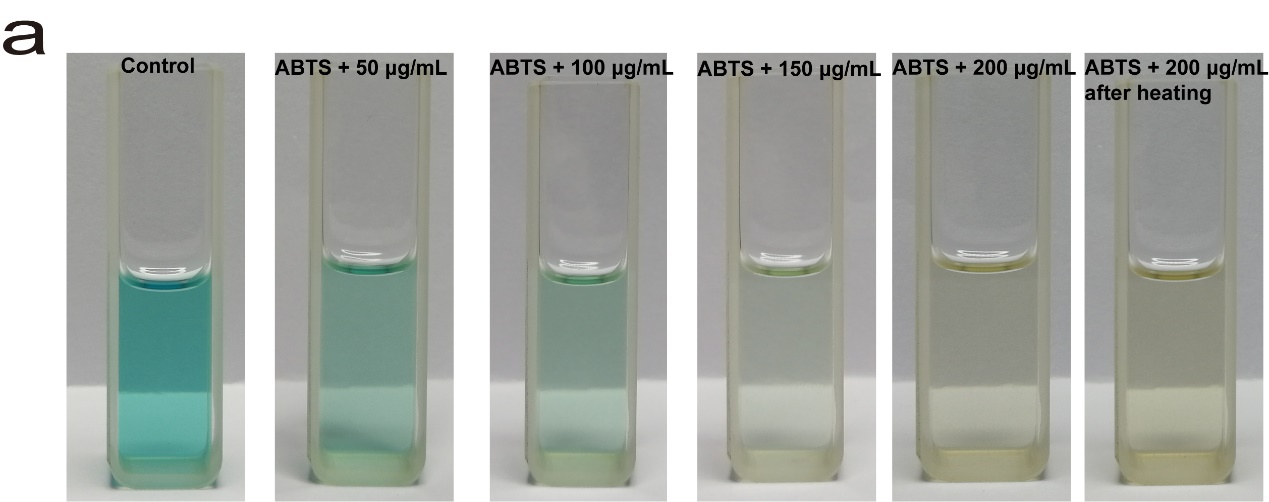


**Figure S2.** (a) The photographs of scavenging ability of MoSe_2_-PVP NPs for ABTS^+^.

**
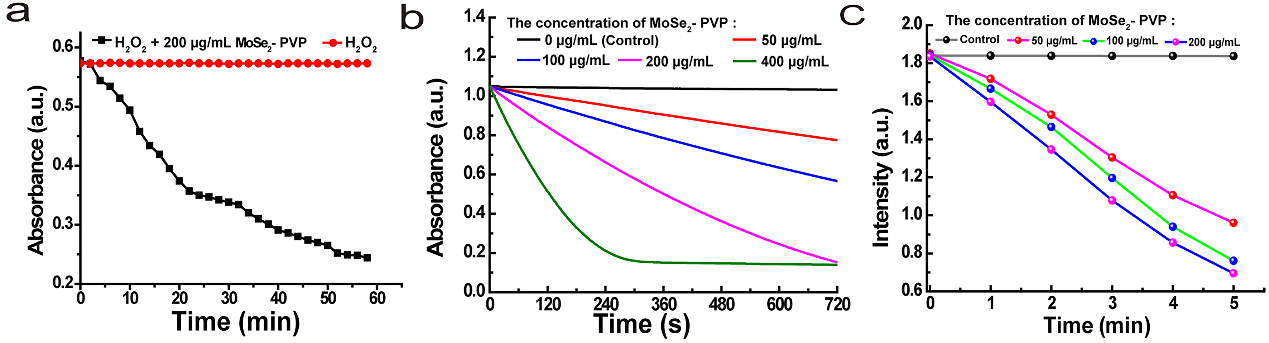
**

**Figure S3.** (a) Absorbance of the reaction of H_2_O_2_ in the presence of MoSe_2_-PVP NPs (200 μg/mL) with time; (b) Kinetic change at 650 nm of POD-like of MoSe_2_-PVP NPs. (c) Kinetic changes at 340 nm of GPx-like of MoSe_2_-PVP NPs.


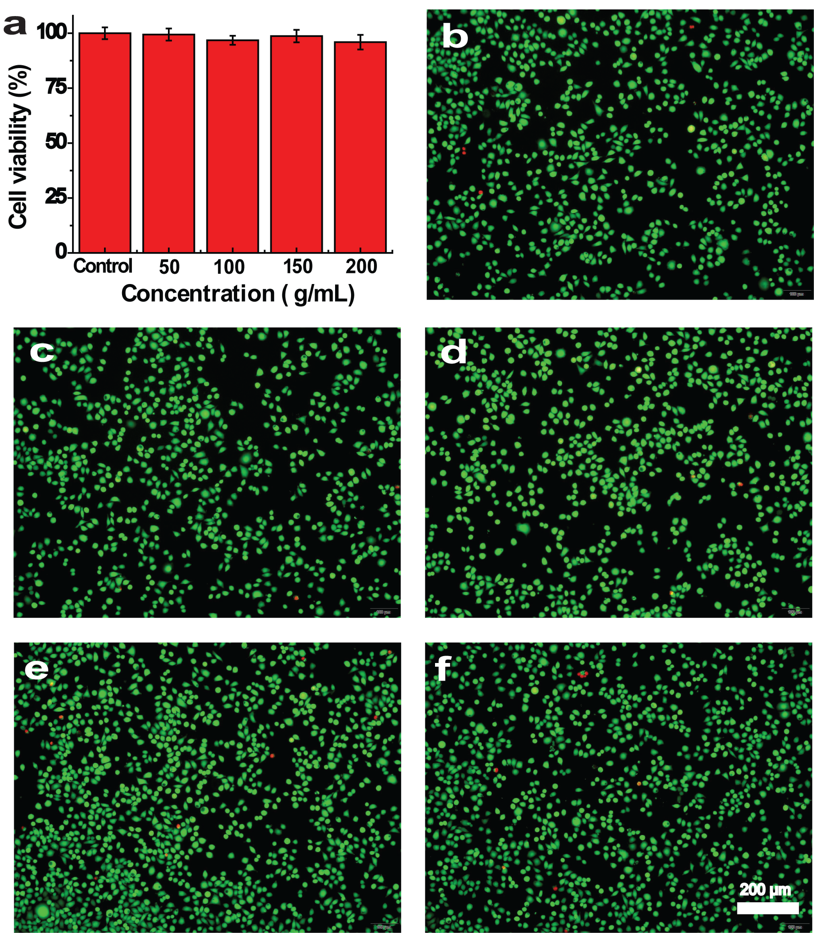


**Figure S4.** The safety of MoSe_2_-PVP NPs in vitro. (a) Cells viabilities of 266-6 cells after cultured with different concentrations of MoSe_2_-PVP NPs. (b) The photographs of Live/Dead staining corresponding to (a), b: control, c: 50 μg/mL, d: 100 μg/mL. Representative graphics are shown, n = 3 independent experiments.


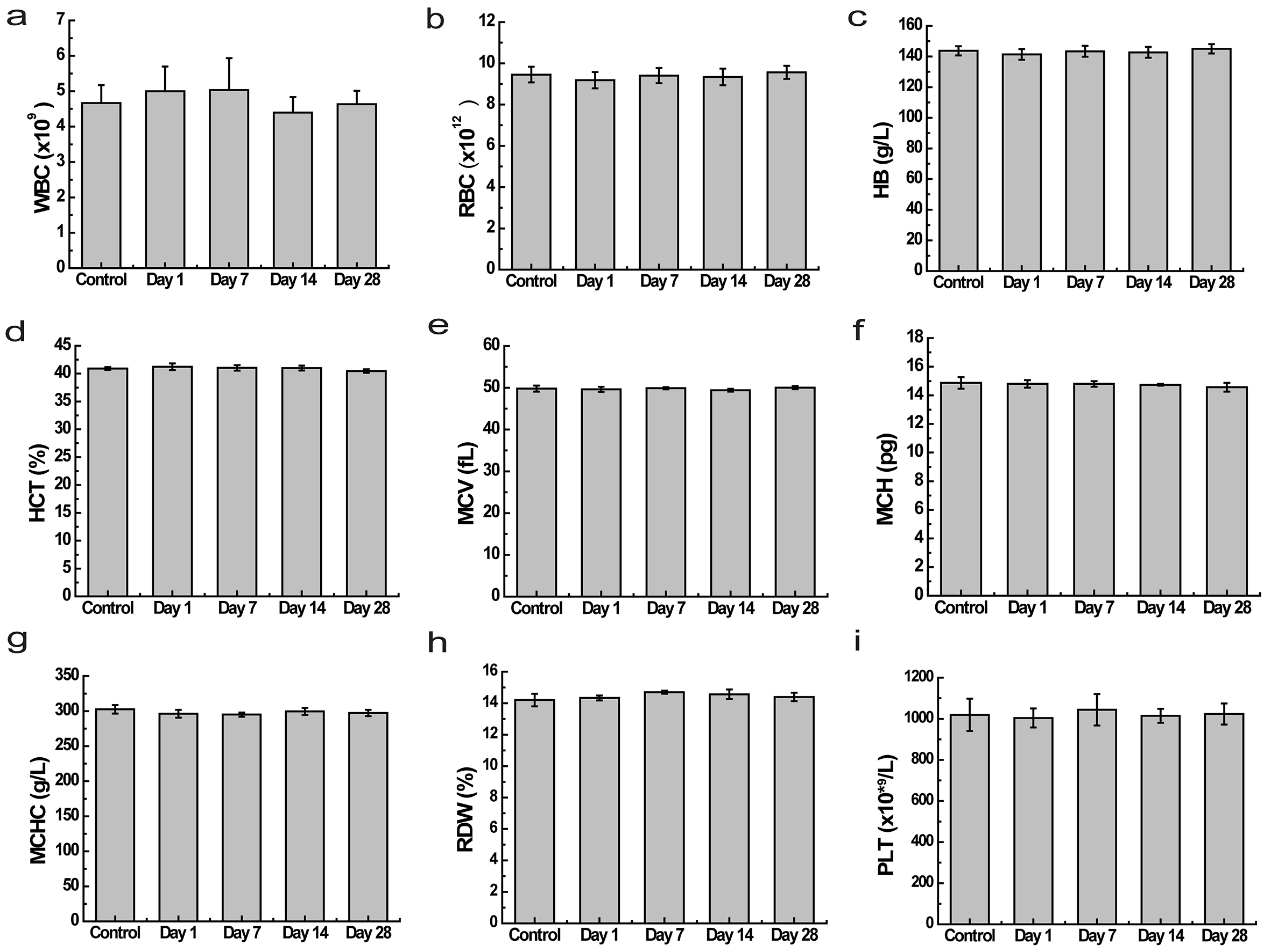


**Figure S5.** (a-i) Hematological test results of mice after the injection with MoSe_2_-PVP NPs.
